# Sleep parameter characteristics of patients with OSA who have retropalatal circumferential narrowing and the clinical significance of lateral pharyngeal wall collapse during sleep

**DOI:** 10.1007/s11325-023-02808-1

**Published:** 2023-03-24

**Authors:** Sun A Han, Hyunkyung Cha, Seung Koo Yang, Seo Young Kim, Doo Hee Han, Dong-Young Kim, Chae-Seo Rhee, Hyun Jik Kim

**Affiliations:** 1 Department of Otorhinolaryngology-Head and Neck Surgery, Hallym University Dongtan Sacred Heart Hospital, Hwaseong-si, Gyeonggi-do, 18450, Korea; 2 Department of Otorhinolaryngology, Seoul National University College of Medicine, Seoul National University Hospital, 103 Daehak-Ro, Jongno-Gu, Seoul, 03080 Korea

**Keywords:** Obstructive sleep apnea, Retropalatal circumferential narrowing, Lateral pharyngeal collapse, Sleep parameters

## Abstract

**Background:**

The lateral pharyngeal wall (LPW) is a critical anatomic structure in patients with obstructive sleep apnea (OSA). Resolving the retropalatal circumferential (RC) narrowing caused by combination of both LPW collapse and antero-posterior (AP) narrowing holds promise for surgical treatment of OSA. We sought to determine the clinical characteristics and distinctive alterations in sleep parameters of patients with OSA who have RC narrowing and LPW collapse.

**Methods:**

Drug-induced sleep endoscopy (DISE), polysomnography findings, and sleep questionnaires were reviewed retrospectively in patients with OSA.

**Results:**

Of the 106 OSA patients examined, 48% showed RC narrowing and 44% showed AP narrowing at the oropharynx level during sleep while 8% of the patients showed only LPW collapse. Patients with RC narrowing with LPW collapse exhibited a higher BMI than those with AP narrowing only. In addition, patients with RC narrowing showed more aggravated sleep parameters including apneic events than patients with AP narrowing alone. The degree of RC narrowing correlated significantly with the severity of OSA as shown by a higher apnea index and lower oxygen desaturations.

**Conclusions:**

Our clinical findings suggest that the presence of RC narrowing with LPW collapse in OSA is closely related to increased apneic and oxygen desaturation events. RC narrowing with LPW collapse may be targets for surgical correction in patients with OSA to improve therapeutic outcomes.

## Introduction

Clinically, obstructive sleep apnea (OSA) occurs due to fixed or dynamic upper airway narrowing caused by abnormal alterations of anatomic structures that limit the natural airflow from the nasal cavity to the hypopharynx during sleep. Excessive narrowing of the upper airway increases negative pressure in the pharyngeal airway during sleep and predisposes the pharynx to become more collapsible[1, 2]. Both upper airway narrowing and increased airway resistance reportedly contribute to the underlying pathogenesis of OSA, which causes symptoms and systemic complications if not properly treated [3–8]. Upper airway obstruction in OSA can be caused by collapse at multiple levels, such as the soft palate, uvula, palatine tonsils, lateral pharyngeal walls, tongue base and supraglottis [9, 10]. The soft palate is the most frequent site of collapse in OSA, and numerous surgical techniques have been designed to modify palatal anatomy [11]. Palatal surgeries for OSA aim to correct the excessive pharyngeal narrowing caused by abnormal changes in anatomic structures and to enhance the tension of the pharyngeal muscles in order to widen the pharyngeal lumen.

The lateral pharyngeal wall (LPW) is a complex structure composed of numerous pharyngeal muscle groups, such as the palatopharyngeus, superior pharyngeal constrictor, and palatoglossus muscles, in addition to lymphoid tissue, including the palatine tonsils. Retropalatal circumferential (RC) narrowing due to LPW collapse has been documented as a critical structural cause of OSA [11, 12]. Recent clinical studies suggest that the LPW might be more collapsible or thicker in patients with severe OSA than in healthy volunteers or patients with mild OSA [13, 14]. Complete RC narrowing might be closely related to higher apnea–hypopnea index (AHI) scores, and higher lateral pharyngeal collapsibility is seen in patients who show a relapse of snoring or apneic events after palatal surgery [15–18]. [15–18]. However, commonly attempted palatal surgeries do not address the lateral pharyngeal wall and do not routinely include the procedures for correction of RC narrowing. Surgical approaches that improve RC narrowing and maintain pharyngeal tension in the lateral dimensions appear to be important for patients with LPW collapse in the upper airway [15–17, 19]. Therefore, before starting treatment or deciding on a therapeutic option for OSA, it is necessary to evaluate whether RC narrowing is present and determine its extent. However, the clinical significance of RC narrowing and the characteristic sleep parameters related to circumferential narrowing at the level of the palate are underestimated when evaluating OSA. Drug-induced sleep endoscopy (DISE) is crucial to identify the presence and degree of RC narrowing, and correlating these findings with sleep parameters is paramount.

In this study, we compared the clinical characteristics of patients with RC narrowing with those of patients with antero-posterior (AP) narrowing or only LPW collapse. We further evaluated the differences in sleep parameters based on the degree of narrowing. Our study highlights the clinical significance of RC narrowing in OSA and provides information about the need to correct AP narrowing and LPW collapse simultaneously in OSA to maintain upper airway stability during sleep.

## Methods

### Patients and study design

Patients diagnosed with OSA at Seoul National University Hospital from March 2017 to December 2021 were recruited, and their upper airway narrowing was confirmed through DISE. This retrospective study was approved by the Institutional Review Board of Seoul National University Hospital (2206–081-1332) and the requirement for informed consent was waived. OSA was diagnosed using level 1 full-time polysomnography (PSG, Grael 4 K, Compumedics, Victoria, Australia), and the severity of OSA was defined as mild for AHI ≥ 5/hr and < 15/hr, moderate for AHI ≥ 15/hr and < 30/hr, and severe for AHI ≥ 30/hr [20]. The medical records, including the PSG and DISE findings were reviewed retrospectively. Sleep parameters such as AHI (events/hr), respiratory distress index (RDI, events/hr), respiratory event–related apnea (RERA, events/hr), apnea index (AI, events/hr), and O_2_ saturation related parameters were examined. The Epworth Sleeping Scale (ESS), Pittsburgh Sleep Quality Index (PSQI), and Beck Depression Inventory (BDI) were completed at discharge following PSG.

### DISE

DISE was performed as part of our standard assessment for OSA [21]. Briefly, sleep was induced by intravenous administration of midazolam (initial dose of 3 mg for adult patients over 50 kg; 0.06 mg/kg). Once the patient had achieved an adequate sleep status, a flexible fiberoptic endoscope was introduced. Obstruction was assessed during desaturation events (decrease of greater than 3% of basal saturation during sleep), and representative findings were recorded. If no desaturation events occurred, changes were analyzed during snoring. An additional bolus of 0.5 mg of midazolam was administered in the event of awakenings. DISE findings were classified according to the VOTE classification [22]. Based on DISE findings, RC narrowing was defined as narrowing of the palate in AP direction combined with LPW collapse. The degree of obstruction was determined as 0, no obstruction; 1, partial obstruction (vibration with desaturation); and 2, complete obstruction (total collapse of airway with desaturation). Degree of snoring was determined by the sleep surgeon performing DISE based on an arbitrary scale used at our center: I, no snoring; II, mild; III, mild to moderate; IV, moderate; V, moderate to severe; IV, severe.

### Statistical analysis

Statistical calculations were performed using SPSS 19.0 (SPSS, IBM). Continuous variables were compared between groups using t-test, Mann Whitney U-test and categorical variables were compared between groups using Pearson Chi-Square or Fisher’s exact test. Differences were considered significant when *p* < 0.05. To analyze the relationship between sleep parameters and OSA severity determined by AHI according to obstruction types, multiple regression analyses were performed.

## Results

### Comparison of clinical characteristics in RC narrowing vs. AP collapse

**Fig. 1:**
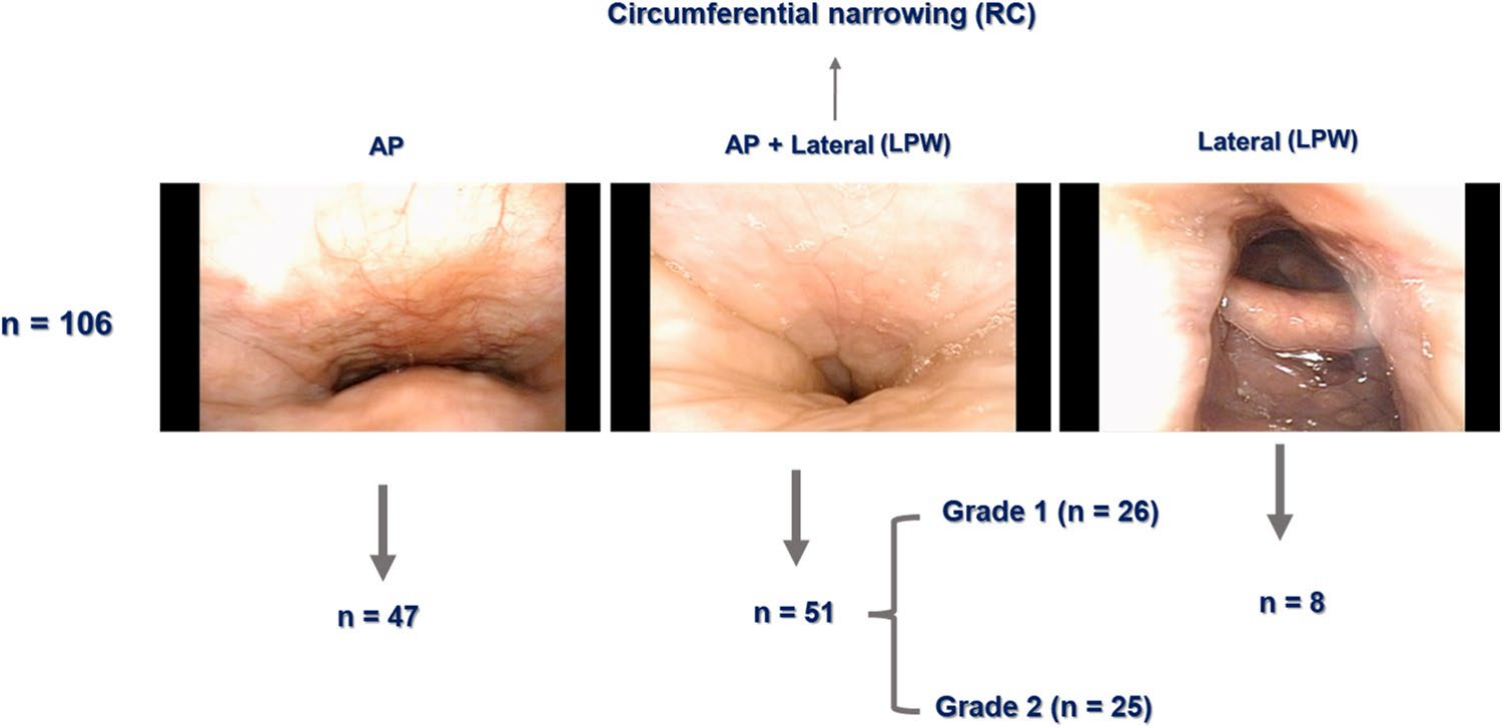
Schematic of the classification of patients depending on DISE findings

Clinical characteristics of patients with RC narrowing and those with only AP narrowing were compared. DISE findings show that 44% of patients (N = 47) exhibited AP narrowing, and 48% (n = 51) had circumferential narrowing at the level of the soft palate. LPW collapse without AP narrowing was observed in just 8 patients. Twenty-six patients with RC narrowing had grade 2 narrowing, while 25 patients had grade 3 narrowing (Fig. 1). The mean age of the patients with RC narrowing was 48.2 years (range, 20–54 years), and 94% were male. Their mean body weight was 82.2 kg, average height was 171 cm, and their mean body mass index (BMI) was 28.2 kg/m^2^. No difference was found in the mean age, height, or sex ratio among patients with RC narrowing and those with AP narrowing. However, both mean body weight and BMI of patients with RC narrowing were significantly higher than thosewith AP narrowing only (Table 1).

**Table 1:** Demographic information of patients with RC and AP narrowing (n = 98)

	RC Narrowing (*n* = 51)	AP narrowing (*n* = 47)	*p*-value
Sex (M:F)	48: 3	43: 4	0.707
Age (years)	48.9 (± 13.2)	50.2 (± 14.4)	0.640
Weight (kg)	82.2 (± 11.5)	75.1 (± 14.2)	**0.009**
Height (cm)	170.9 (± 6.6)	168.9 (± 8.4)	0.182
BMI (kg/m^2^)	28.2 (± 3.4)	26.2 (± 3.7)	**0.007**

RC, retropalatal circumferential; AP, antero-posterior; BMI, Body mass index. Statistics performed by t-test. *p*-values in bold indicate those that are statistically significant

**Fig. 2:**
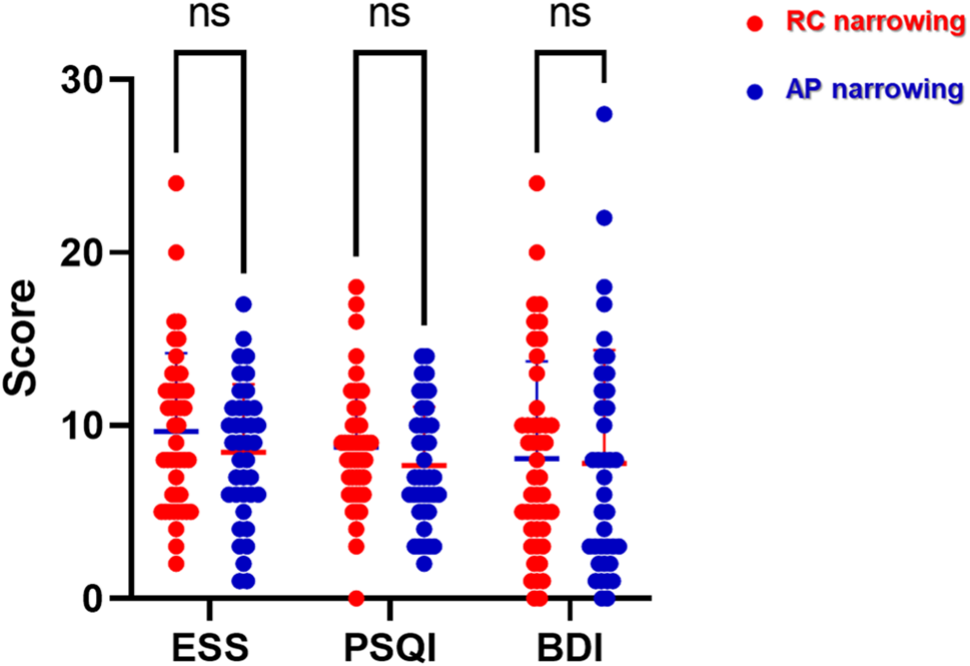
Comparison of subjective symptoms of patients based on sleep questionnaires, Comparison of Epworth Sleepiness Scale (ESS), Pittsburgh Sleep Quality Index (PSQI), and Beck Depression Inventory (BDI) between patients with RC narrowing (red circle) and those with AP narrowing (blue circle). Statistics performed by t-test, * *p* < .05

Scores of clinical questionnaires about subjective sleep-related symptoms were examined to compare differences between patients with RC narrowing and those with AP narrowing. Our questionnaire results show that the ESS and PSQI scores of patients with RC narrowing were higher than those of patients with AP narrowing, whereas the BDI scores in the two groups were similar (Fig. 2). These clinical data show that patients with RC narrowing showed a relatively higher BMI and more severe subjective symptoms, including daytime sleepiness.

### Comparison of sleep parameters in RC narrowing vs. AP collapse

**Fig. 3:**
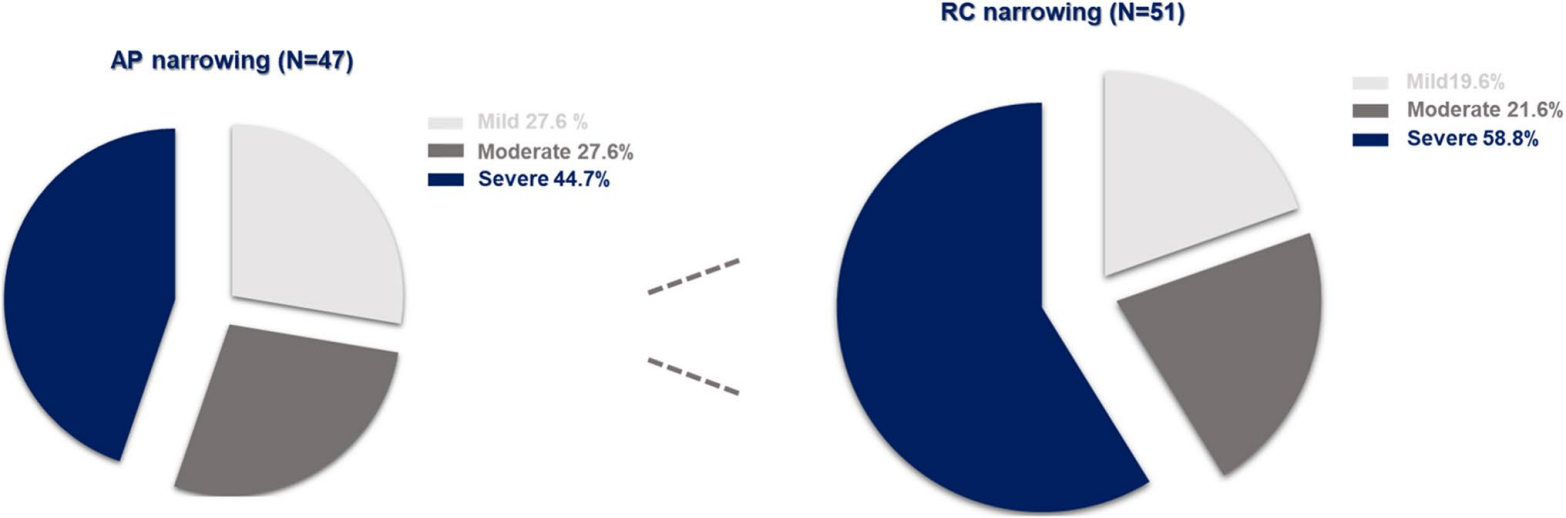
Comparison of OSA severity based on AHI score. The severity of OSA was compared in patients with AP (Lt) and RC narrowing (Rt).

Our PSG data showed that 28% of patients with AP narrowing had moderate OSA, and 45% of them had severe OSA. On the other hand, 22% of patients with RC narrowing had moderate OSA, and the proportion of patients with severe OSA was higher (59%), although the difference was not statistically significant (Fig. 3).

Polysomnography parameters between patients with RC narrowing and those with AP narrowing were compared. The mean AHI score was 41.2 ± 27.3 (events/hr) in patients with RC narrowing, which was significantly higher than that of patients with AP narrowing (30.7 ± 20.9 (events/hr); *p* = 0.036) (Table 2). In addition, supine AHI, non-supine AHI, and REM sleep AHI scores were all significantly higher in patients with RC narrowing (*p* = 0.048, *p* = 0.002, and *p* = 0.006, respectively). Although no statistical significance was observed in other sleep parameters, indexes related to arousals were relatively higher, and values related to oxygen saturation were relatively lower in patients with RC narrowing than those with AP narrowing.

**Table 2:** Sleep parameters in patients depending on RC and AP narrowing during sleep

	RC narrowing (*n* = 51)	AP narrowing (*n* = 47)	*p*-value
AHI (event/hr)	41.2 (± 27.3)	30.7 (± 20.9)	**0.036**
AI (event/hr)	23.3 (± 26.9)	16.7 (± 20.1)	0.160
HI (event/hr)	17.9 (± 13.3)	14.0 (± 9.9)	0.139
AHI (supine) (event/hr)	54.8 (± 26.2)	42.7 (± 20.9)	**0.048**
AHI (non-supine) (event/hr)	32.2 (± 37.7)	10.8 (± 13.8)	**0.002**
AHI (REM) (event/hr)	39.2 (± 28.4)	29.2 (± 19.7)	**0.006**
AHI (NREM) (event/hr)	39.2 (± 28.4)	29.6 (± 22.3)	0.067
Longest apnea (sec)	46.4 (± 18.8)	50.2 (± 37.6)	0.580
Longest hypopnea (sec)	46.2 (± 23.6)	59.0 (± 61.6)	0.842
RERA (event/hr)	0.12 (± 0.33)	0.16 (± 0.34)	0.557
RDI (event/hr)	41.3 (± 27.3)	30.8 (± 20.9)	**0.036**
Arousal events	12.5 (± 23.1)	6.8 (± 15.1)	0.486
Arousal Index (event/hr)	1.93 (± 3.74)	1.04 (± 2.45)	0.467
Average SpO2 (%)	91.6 (± 4.6)	91.6 (± 7.0)	0.356
Minimum SpO2 (%)	76.2 (± 10.7)	79.3 (± 10.8)	0.159
% of sleep SpO2 < 90% (%)	10.6 (± 16.1)	8.9 (± 17.5)	0.608

Abbreviations: RC, retropalatal circumferential; AP, antero-posterior; AHI, Apnea–Hypopnea Index; AI, Apnea Index; HI, Hypopnea Index; REM, Rapid eye movement; NREM, Non-rapid eye movement; RERA, Respiratory Effort Related Arousal; RDI, Respiratory Disturbance Index. Statistics performed by t-test. *p*-values in bold indicate those that are statistically significant

Multiple linear regression was performed to test if polysomnography parameters significantly predicted OSA severity as determined by AHI. In patients with RC narrowing, the fitted regression model was: AHI = 375.128–4.217*average SpO2 + 1.849*BMI (R^2^ = 0.842, F = 58.556, *p* < 0.001). Lower average SpO2 significantly predicted OSA severity (ß = -4.217, *p* < 0.001). Furthermore, BMI also significantly predicted OSA severity in patients with RC narrowing (ß = 1.849, *p* = 0.012). In patients with AP narrowing, the fitted regression model was: AHI = 364.039–3.606*average SpO2 (R^2^ = 0.814, F = 78.658, *p* < 0.001). Lower average SpO2 was the only significant predictor of increased AHI (ß = -3.606, *p* < 0.001).

These results support the hypothesis that patients with RC narrowing exhibit more aggravated sleep parameters than patients with AP narrowing, which could be associated with an increase in OSA severity.

### Comparison of clinical characteristics and polysomnography parameters in RC narrowing according to the degree of narrowing

**Fig. 4:**
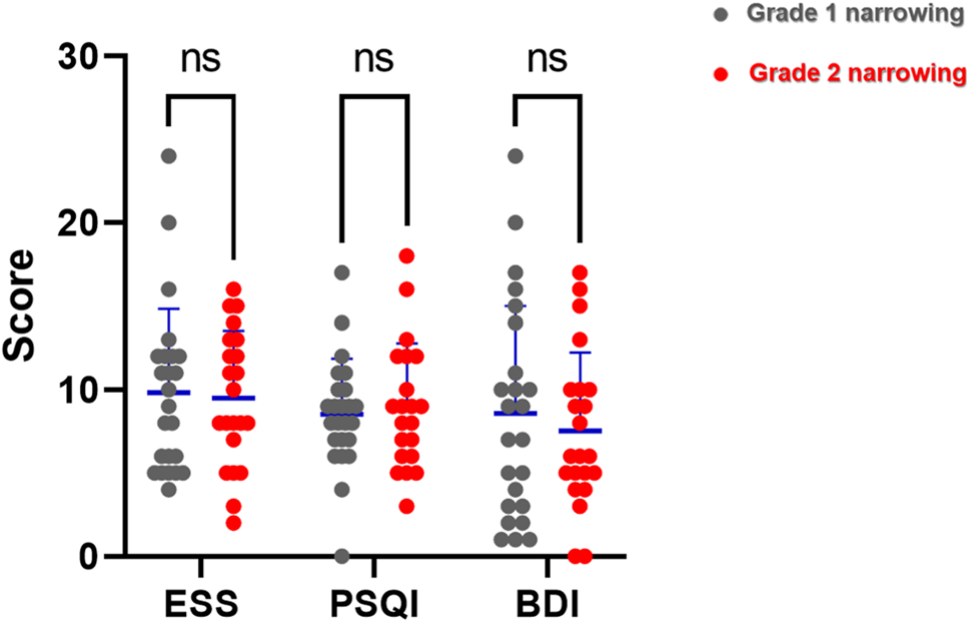
Comparison of subjective symptoms in patients with RC narrowing, based on the sleep questionnaires. Comparison of Epworth Sleepiness Scale (ESS), Pittsburgh Sleep Quality Index (PSQI), and Beck Depression Inventory (BDI) between patients with grade 2 (red circle) and grade 3 RC narrowing (blue circle). Statistics performed by Mann Whitney U-test., * *p* < .05

Among the 51 patients confirmed to have RC narrowing during sleep, 26 were confirmed to have grade 1 RC narrowing, and 25 were classified into grade 2. No statistically significant differences were observed between those two groups in sex, weight, or BMI. However, the mean age (52.4 years old) of patients with grade 2 RC was higher than that of those with grade 1 narrowing (45.5 years old), although statistical significance was not reached (*p* = 0.060) (Table 3). We found that subjective symptoms of patients with RC narrowing did not vary significantly with the degree of narrowing (Fig. 4). However, the polysomnography parameters were significantly higher in patients with higher degree of obstruction. The mean AHI was 51.7 ± 26.3 (events/hr) in patients with grade 2 RC narrowing, while it was 31.1 ± 24.7 (events/hr) in those with grade 1 narrowing (*p* = 0.006) (Table 4). Furthermore, supine AHI, and NREM AHI scores were all significantly elevated in patients with grade 2 RC narrowing (*p* = 0.001 and *p* = 0.007, respectively). The longest apnea time was significantly longer in patients with grade 2 RC narrowing than in those with grade 1 RC narrowing (p < 0.001) (Table 4). Similar to AHI results, the mean RDI was significantly higher in patients with grade 2 narrowing (*p* < 0.006). Values related to sleep oxygen saturation (average SpO2, minimum SpO2, and % of sleep SpO2 < 90%) were significantly lower in patients with grade 2 RC narrowing (*p* < 0.001, *p* = 0.002, and *p* < 0.001, respectively). The current findings indicate that polysomnography parameters related to apneic events and hypoxemia become more aggravated according to the degree of oropharyngeal obstruction during sleep in patients with RC narrowing.

**Table 3:** Demographic information of recruited OSA subjects with RC narrowing (n = 51)

	Grade 1 RC narrowing (*n* = 26)	Grade 2 RC narrowing (*n* = 25)	*p*-value
Sex (M:F)	24: 2	24: 1	> 0.999
Age (years)	45.5 (± 14.7)	52.4 (± 10.6)	0.060
Weight (kg)	80.2 (± 12.2)	84.0 (± 10.5)	0.242
Height (cm)	171.0 (± 6.6)	170.8 (± 6.6)	0.915
BMI (kg/m^2^)	27.4 (± 3.7)	29.0 (± 3.0)	0.102

RC, retropalatal circumferential; AP, antero-posterior; BMI, body mass index. Statistics performed by Mann Whitney U-test

**Table 4:** Sleep parameters in OSA subjects with RC narrowing depending on the degree

	Grade 1 RC narrowing (*n* = 26)	Grade 2 RC narrowing (*n* = 25)	*p*-value
AHI (event/hr)	31.1 (± 24.7)	51.7 (± 26.3)	**0.006**
AI (event/hr)	11.2 (± 14.3)	35.9 (± 31.1)	** < 0.001**
HI (event/hr)	19.9 (± 15.5)	15.8 (± 10.6)	0.281
AHI (supine) (event/hr)	41.5 (± 26.9)	67.4 (± 18.3)	**0.001**
AHI (nonsupine) (event/hr)	24.1 (± 27.6)	39.9 (± 44.7)	0.163
AHI (REM) (event/hr)	37.2 (± 24.2)	44.9 (± 18.4)	0.211
AHI (NREM) (event/hr)	29.0 (± 26.0)	49.8 (± 27.2)	**0.007**
Longest apnea (sec)	36.8 (± 15.1)	56.4 (± 17.1)	** < 0.001**
Longest hypopnea (sec)	43.5 (± 14.7)	49 (± 30.4)	0.952
RERA (event/hr)	0.12 (± 0.24)	0.12 (± 0.41)	0.239
RDI (event/hr)	31.2 (± 24.7)	51.8 (± 26.2)	**0.006**
Arousal events	21.5 (± 28.2)	3.0 (± 10.0)	**0.002**
Arousal Index (event/hr)	3.3 (± 4.6)	0.5 (± 1.9)	**0.002**
Average SpO2 (%)	93.7 (± 2.4)	89.4 (± 5.3)	** < 0.001**
Minimum SpO2 (%)	80.6 (± 8.6)	71.7 (± 10.9)	**0.002**
% of sleep SpO2 < 90%	3.4 (± 7.0)	18.2 (± 19.3)	** < 0.001**

Abbreviations: RC, retropalatal circumferential; AHI, Apnea–Hypopnea Index; AI, Apnea Index; HI, Hypopnea Index; REM, Rapid eye movement; NREM, Non-rapid eye movement; RERA, Respiratory Effort Related Arousal; RDI, Respiratory Disturbance Index. Statistics performed by Mann Whitney U-test. *p*-values in bold indicate those that are statistically significant

### Polysomnography parameters in LPW collapse only

**Fig. 5:**
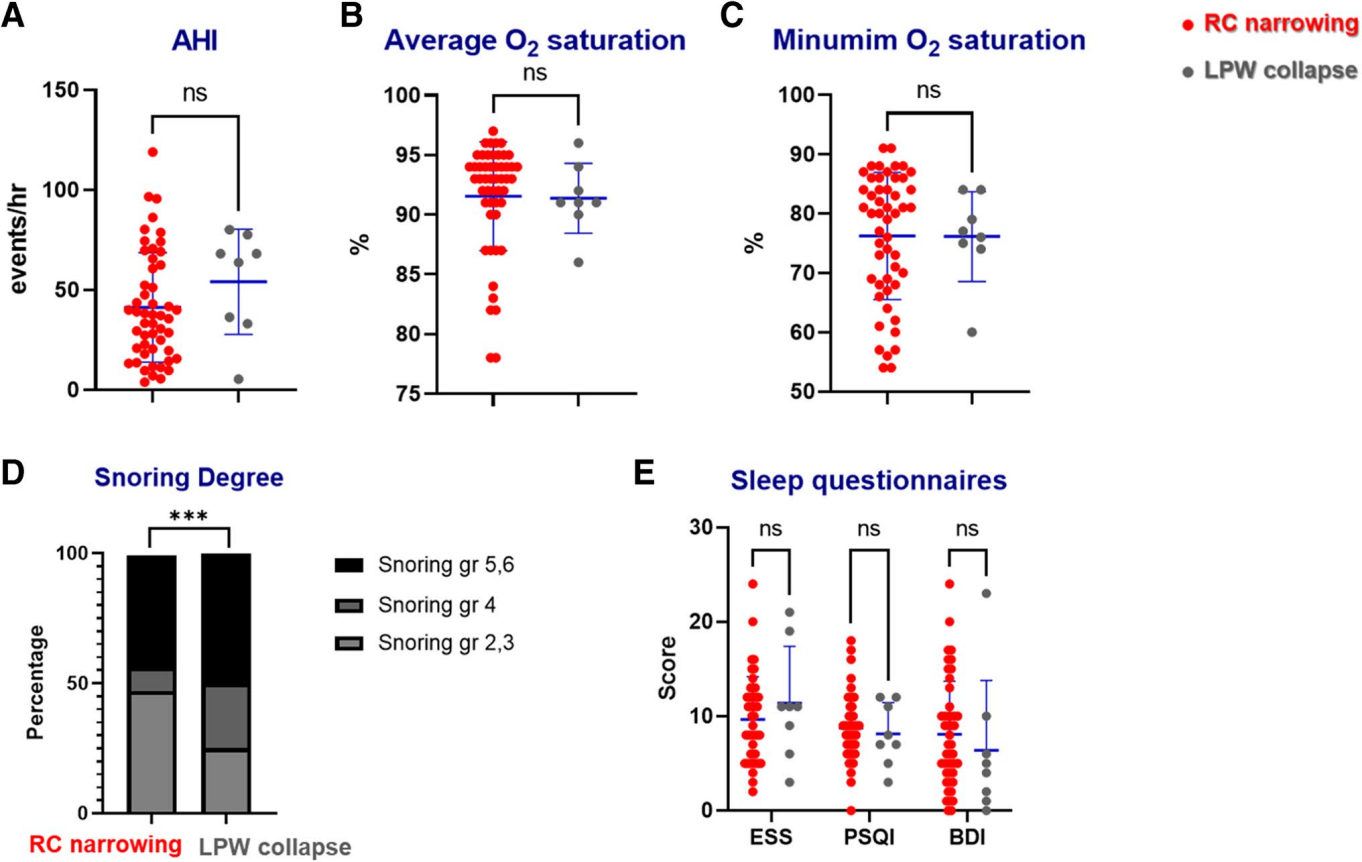
Comparison of sleep parameters and sleep questionnaires between patients with RC narrowing and those with LPW collapse. (A) Apnea and hypopnea index, (B) average O2 saturation, and (C) lowest oxygen saturation during sleep were compared between patients with RC narrowing and those with LPW collapse. (D) The intensity of snoring was compared between patients with RC narrowing and those with LPW collapse. Patients with RC narrowing and those with LPW collapse completed the (E) Epworth Sleepiness Scale (ESS), Pittsburgh Sleep Quality Index (PSQI), and Beck Depression Inventory (BDI) (VAS: visual analogue scale). *: *p* < .05 between patients with RC narrowing (gray circle) and those with LPW collapse (red circle).

Among the 106 patients with OSA, 8 were confirmed to have only LPW collapse. The mean BMI of the patients with only LPW collapse was 27.8 ± 3.5 kg/cm^2^ and the mean AHI was 50.2 (events/hr), which was higher than patients with RC narrowing, but that difference was not statistically significant (Fig. 5A). In addition, neither the average nor minimal O2 saturation differed between patients with RC narrowing and those with only LPW collapse (Fig. 5B, 5C). Furthermore, the proportion of patients with loud snoring was higher in those with LPW collapse only (Fig. 5D). Specifically, 75% of the patients with only LPW collapse during sleep exhibited greater than moderate degree (grade IV) of snoring, whereas only 53% of the patients in the RC narrowing group exhibited more than moderate degree of snoring. 48% of patients with RC narrowing showed low intensity snoring during sleep. The PSQI and BDI scores tended to be lower in patients with LPW collapse compared to those with RC narrowing; however, the difference was not statistically significant (Fig. 5E). Although more powered tests for direct comparison are warranted, sleep parameters and subjective sleep-related symptoms of patients with only LPW collapse may not differ greatly with RC narrowing except the intensity of snoring.

## Discussion

In this study, patients with RC narrowing exhibited more aggravated sleep parameters than patients with AP narrowing. Various obstructive patterns appear in the soft palate and oropharynx of patients with OSA. Our data highlight the clinical significance of RC narrowing, as patients with this pattern of obstruction showed increased OSA severity.

The LPW is more collapsible when pressured by airflow in patients with OSA than in healthy patients, and the LPW of patients with OSA can be thicker than that in normals , making it a predominant anatomic factor in airway narrowing in OSA [12, 15]. As stated before, LPW collapse in OSA contributes to increased airway resistance, causing partial or complete RC narrowing during sleep [16, 17, 23–25]. It has been verified that LPW collapse-related airway resistance causes aggravated intermittent hypoxia and oxygen desaturation in OSA [26]. In addition, OSA with RC narrowing exhibits higher AHI and RDI scores than OSA with AP narrowing only [12, 15, 27]. Therefore, an adequate therapeutic option is needed to improve RC narrowing in the treatment of OSA. In particular, the clinical confirmation of RC narrowing is essential to determine the appropriate therapeutic option, such as those increasing the maintenance of tension or stability in the LPW, combined with correction of AP narrowing [23–25].

Our clinical data also revealed that OSA with RC narrowing had markedly higher AHI, and RDI than that of OSA with only AP narrowing. In addition, the BMI of patients with RC narrowing was significantly higher than in the group with AP narrowing. Multiple regression analysis showed that low average SpO2 and higher BMI were significant risk factors for increased OSA severity in patients with RC narrowing while only lower average SpO2 was a significant risk factor in those with AP narrowing. Thus, the existence of RC narrowing may be related to high BMI and can cause more frequent airway obstruction during sleep, resulting in increased apneic events. Our study added evidence that it is important to determine the obstructive pattern of oropharynx in OSA before choosing a therapeutic option.

In previous studies that emphasized the clinical significance of RC narrowing in OSA, parameters indicative of hypoxemia during sleep, such as ODI, time of SpO_2_ < 90%, and minimal SaO_2_, correlated significantly with complete LPW collapse [26, 28]. Those findings are of great significance because hypoxemia plays a critical role in the pathophysiology of OSA-related cardiovascular complications, and repeated decreases in blood oxygen during sleep in OSA have been postulated to have several metabolic consequences [26, 29]. Our findings suggest that, as disease severity increases, treatments that address RC narrowing of the upper airway may be considered to prevent the serious complications derived from repeated hypoxemia. In contrast to other recent reports, our clinical data show that hypoxemia-related parameters during sleep did not differ significantly between patients with RC narrowing and those with AP narrowing, whereas sleep parameters related to apneic events were aggravated in patients with RC narrowing. Previous studies have suggested that AHI may not always correlate with symptoms of patients with OSA and even in patients with similar AHI the parameters related to oxygenation may differ [30, 31]. AHI and RDI were significantly higher in patients who had complete RC narrowing than in those with partial narrowing, and more aggravated sleep parameters were also observed in patients with grade 2 RC narrowing, depending on sleep position and sleep stage. Furthermore, both the average SpO_2_ (%) and minimal SpO_2_ (%) were significantly lower in patients with grade 2 RC narrowing, but neither body weight nor BMI differed significantly between patients with grade 1 and grade 2 RC narrowing. It is noteworthy that the presence of RC narrowing might be closely related to a higher frequency of apneic events and greater severity of OSA without affecting hypoxemia during sleep. Nonetheless, patients who exhibit complete RC narrowing may experience significant systemic hypoxemia during their apneic events. Although a powered comparison could not be made, we postulate that LPW collapse alone may not increase the severity of OSA as much as RC narrowing. However, the LPW collapse is an important component of RC narrowing that needs to be corrected even in patients with LPW collapse alone.

Novel assessments of upper airway narrowing are generally implemented during sleep, and DISE is the most precise modality for predicting events in the oropharynx/lateral wall area and providing information critical for making decisions about therapeutic options for patients with OSA. Therefore, careful attention must be paid to addressing RC narrowing in patients when planning for the targeted management of OSA based on DISE findings.

The biggest limitation of our study is that subjective opinions are required to determine whether or not a patients’s DISE findings indicate LPW collapse and interpret the grade of RC narrowing. Because DISE is subjective, it is inherently prone to inconsistencies, and factors such as the experience of the performing surgeon, depth of sedation, and drug used for sedation can influence the findings. All DISE results in this study were evaluated by an experienced sleep surgeon to minimize that limitation. Furthermore, the DISE in this study was performed without any blinding, and the author rating the videos was not blinded to the sleep study variables. Therefore, the interpretation of DISE findings is partially subjective, and the results in determining RC narrowing and LPW collapse may be biased in this study. Another limitation of the study is that midazolam was used for sedation in DISE. Although midazolam was the drug of choice according to our institution’s protocol, it has been suggested that midazolam may cause more tongue base collapse compared to other drugs such as dexmedetomidine [32].

## Conclusions

Our clinical findings suggest that RC narrowing in the oropharynx of patients with OSA may be closely related to the aggravation of OSA severity compared to AP narrowing. This study deepens the understanding about the significance of LPW collapse especially in patients with RC narrowing and that adequate treatment options should be selected depending on obstructive patterns to improve the therapeutic outcome.

## Data Availability

Not applicable.
